# Impact of reduction in contact time activity of infected individuals on the dynamics and control of directly transmitted respiratory infections in SIR models

**DOI:** 10.1186/s13662-020-02708-8

**Published:** 2020-05-27

**Authors:** Muntaser Safan

**Affiliations:** 1 Mathematics Department, Faculty of Science, Mansoura University, Mansoura, Egypt; 2 Department of Mathematical Sciences, Faculty of Applied Science, Umm Al-Qura University, Makkah, Saudi Arabia

**Keywords:** 92Bxx, Herd immunity, Equilibria, Stability analysis, Maximum treatment capacity, Hopf bifurcation

## Abstract

This paper aims to study the impact of using an educational strategy on reducing the efforts needed to control respiratory transmitted infections represented by SIR models, taking into account heterogeneity in contacts between infected and non-infected individuals. Therefore, a new incidence function, in which the difference in contact time activity between infected and non-infected individuals is taken into account, is formulated. Equilibrium and stability analyses of the model have been carried out. The model has been extended to include the effect of herd immunity and the analysis showed that the higher the percent reduction $\widehat{P}_{r}$ in the contact-activity time of infected individuals is, the lower the critical vaccination coverage level $p_{c}$ required to eliminate the infection is, and therefore, the lower the infection’s minimum elimination effort is. Another extension of the basic model to include a control strategy based on treating infected individuals at rate *α* with a maximum capacity treatment $\mathcal{I}$ has been considered. The equilibrium analysis showed the existence of multiple subcritical and supercritical endemic equilibria, while the stability analysis showed that the model exhibits a Hopf bifurcation. Simulations showed that the higher the maximum treatment capacity $\mathcal{I}$ is, the lower the value of the critical reduction in infected individuals’ time activity $P_{r}^{\star}$, at which a Hopf bifurcation is generated, is. Simulations with parameter values corresponding to the case of influenza A have been carried out.

## Introduction

Epidemiology is often considered the cornerstone of public health. It studies the factors related to population health and helps to find the scientific basis upon which public health decision makers put strategies to prevent and control infectious diseases. In this respect, mathematical epidemiology plays a pivotal role through the formulation, analysis, and deployment of mathematical models describing the spread of the disease of concern [1, 2, 25].

Mathematical models have been extensively used to help extend our understanding of the transmission dynamics and controllability of infectious diseases both on the micro- and macro-scales [9, 10, 20, 24]. Infectious diseases are transmitted directly (e.g. influenza, measles, smallpox, etc.) and/or indirectly (e.g. cholera, dengue, malaria, Leishminiasis, etc.). An important term that is crucial while modeling the transmission dynamics of infectious diseases is the incidence term. It describes the dynamics of interaction between susceptible and infected individuals and accounts for the number of new infected cases per unit time.

The literature shows the use of various forms of the incidence function, with general mathematical form $g(I, N) S$, where $S (I)$ denotes the number of susceptible (infected) individuals at time *t*. The term $g(I, N)$ denotes the “*force of infection*” (i.e., the rate at which susceptible individuals acquire the infection). The parameter *N* denotes the total population size at time *t*. Sometimes, the “*force of infection*” is given by $g(I, N) = \beta I^{p} / (1 + a I^{q})$, where *p* and *q* are positive integers and $p \ge q$ (see for example [15] and the references therein). However, mostly, it takes the form $g(I, N) = \beta C(N) I/N$ [10, 28], where *β* is the transmission rate [10] and $C(N)$ is the probability that an individual takes part in a contact [29].

In some papers [1, 5], the incidence term is assumed bilinear (i.e., in mass-action form), where $C(N) = N$, while in others [3, 10, 12], a standard incidence form ($C(N) = 1$) is used. In other cases, the incidence term is assumed to be saturated ($p = q = 1$) [4, 19, 29] or in a Holling-type form (e.g. $p = 1$ and $q = 2$) [26]. Other non-linear incidence forms are also considered in the literature [15]. In this work, we focus on incidence functions of the standard incidence form.

Our work is motivated basically by the potential attack of infectious diseases (represented by SIR models) for which neither vaccine nor treatment is basically in hand. Therefore, self-isolation and self-quarantine are the fundamental strategies used to flatten the curve or to contain the infection, like the case of the pandemic covid-19. The model has then been extended to the case of herd immunity, assuming that a vaccine does exist. Finally, we considered the case of limited treatment supply. In all cases, the model has been thoroughly analyzed and the conditions ensuring an effective control of the infection are mostly obtained. The main difference between our modeling approach and the approaches studied before [1, 3, 10, 22, 25] is the formulation of the incidence function.

Rather than assuming that all individuals have the same availability and desire to contact with others in the same population, a heterogeneity between infected and non-infected individuals is taken into account. More precisely, it is assumed that infected individuals tend to make fewer contacts per unit time than non-infected ones. Therefore, an SIR model with modified standard incidence function in a demographically stationary population is formulated and mathematically analyzed in Sect. 2. The introduced incidence form contains a parameter $P_{r}$ that accounts for the relative reduction in the availability of infected (with respect to non-infected) individuals to make contacts with other ones. This could be thought of as an educational way to reduce the burden of the infection with little cost. The possibility to control the infection with a strategy based on vaccinating a proportion *p* of newborns and the impact of increasing the reduction parameter $P_{r}$ on reducing the critical vaccination coverage level $p_{c}$ (which is required to eliminate the infection) has been studied in Sect. 3. Moreover, Sect. 4 is dedicated to study the possibility to contain the infection with a strategy based on treating infected individuals with a maximum capacity treatment in the presence of the reduction parameter $P_{r}$. A summary and conclusion for the results is in Sect. 5.

## Model building and analysis

To hit the problem, we consider the classical SIR model for a demographically stationary population

1 $$\begin{aligned}& \frac{dS}{dt} = \mu N - \text{\{New incidences\}} - \mu S, \\& \frac{dI}{dt} = \text{\{New incidences\}} - (\gamma+ \mu) I, \\& \frac{dR}{dt} = \gamma I - \mu R, \end{aligned}$$

where *N* is the total population size (assumed constant). The variables $S(t)$, $I(t)$ and $R(t)$ denote to the number of susceptible, infected and recovered individuals, respectively, at time *t*. We have $S(t) + I(t) + R(t) = N$. In this setting, it is assumed that $1/\mu$ is the average life expectancy, while *γ* is the recovery rate of infected individuals. It is assumed further that the population is homogeneously mixed so that every individual has the same chance to make contacts with every other one in the population.

In epidemiology, the most interesting contacts are those occurring between susceptible and infected individuals, resulting in new incidences. The rate of their occurrence is $\lambda(t) S(t)$, where $\lambda(t)$ is the “*force of infection*”. In the standard incidence setting, the “*force of infection*” is given by $\lambda (t) = \beta I / N$, where *β* is the successful contact rate. This formulation is implicitly based on the assumption that individuals are available to make contacts all the time. However, if we consider the fact that individuals tend to stay far from contacts with others for part of their time (e.g., by either staying indoor lonely, sleeping or any other way so that they do not stay mingling), then this formulation of the new incidences could be slightly modified.

Assume that $p_{0}$ ($p_{1}$) represents the probability that a non-infected (infected) individual stays at rest, in the sense that he/she does not make contacts with others. In fact, this probability could be seen as the proportion of time during which an individual avoids making contacts with others. Then $q_{0} = 1- p_{0}$ ($q_{1} = 1 - p_{1}$) is the proportion of time during which a non-infected (an infected) individual mingles with other individuals in the population. Hence, the total number of susceptible individuals who are available to make contacts at time *t* is $q_{0} S(t)$, while that of infected individuals is $q_{1} I(t)$. Hence, the total number of individuals who are available, while a contact is occurring, at time *t* is $q_{0} [S(t) + R(t)] + q_{1} I(t) = q_{0} N(t) - (q_{0} - q_{1}) I(t)$. Thus, the probability that an infected makes a contact with a susceptible individual at time *t* is $q_{0} S(t)/[q_{0} N(t) + (q_{1} - q_{0}) I(t)]$. Thus, the total number of incidences that occur per unit time, when $q_{1} I(t)$ infected individuals are available to contact with susceptible individuals, is given by

2 $$ \text{New incidences} = \beta\times q_{1} I(t) \times \frac{q_{0} S(t) }{q_{0} N(t) + (q_{1} - q_{0}) I(t)}. $$

Based on the fact that infections affect negatively on the contact activity of infected individuals and, therefore, they tend to stay lonely more time than non-infected individuals, we may assume that $q_{1} \le q_{0}$. It is noteworthy that the difference ($q_{0} - q_{1}$) represents the reduction in the proportion of mingling time due to acquiring the infection. Therefore, the percent reduction in the mingling time proportion of infected individuals is $\widehat{P}_{r} = (100 \times P_{r})$, where

3 $$ P_{r} = (q_{0} - q_{1})/q_{0} = 1 - q_{1}/q_{0}. $$

Hence,

4 $$ \text{New incidences} = \beta\times(1-P_{r}) q_{0} I(t) \times\frac{S(t) }{N(t) - P_{r} I(t)}. $$

Thus, our model reads

5 $$\begin{aligned}& \frac{dS}{dt} = \mu N - \frac{(1-P_{r}) q_{0} \beta S(t) I(t)}{N(t) - P_{r} I(t)} - \mu S, \\& \frac{dI}{dt} = \frac{(1-P_{r}) q_{0} \beta S(t) I(t)}{N(t) - P_{r} I(t)} - (\gamma+ \mu) I, \\& \frac{dR}{dt} = \gamma I - \mu R, \end{aligned}$$

with the initial conditions $S(0), I(0), R(0) > 0$ and $S(0) + I(0) + R(0) = N$. Model (5) is defined on the solution set

6 $$ \varOmega= \bigl\{ (S, I, R) \in{\mathbf{R}}_{+}^{3}, 0\le S\le N, 0 \le I \le N, 0\le R \le N, S(t) + I(t) + R(t) = N \bigr\} . $$

It is worth noting that model (5) is applicable to all infections inducing permanent immunity. It could be modified by including infections-induced mortality to describe the dynamics of lethal infections like smallpox, measles and other lethal diseases like coronaviruses, if the latent period is neglected. Also, some researchers use SIR models to describe the dynamics of single influenza A outbreaks. In the following we present a proposition which summarizes results on the main properties of model (5). Its proof is deferred to Appendix A.

### Proposition 1

*The setΩis positively invariant and attracts all solutions in*${\mathbf{R}}_{+}^{3}$. *Moreover*, *for any nonnegative initial conditions*$(S(0), I(0), R(0)) \in\varOmega$, *the solution set*$(S(t), I(t), R(t))$*of the system* (5) *remain positive for all*$t >0$. *Also*, *model* (5) *has a unique solution*.

### Final size epidemic

One of the most important concepts is the final size epidemic, as it shows the proportion of population that experience the infection and get recovered from it at the end of an epidemic. In this concern, the parameters related to the vital dynamics of model (5) are omitted and, therefore, we have

7 $$\begin{gathered} \frac{dS}{dt} = - \frac{(1-P_{r}) q_{0} \beta S(t) I(t)}{N(t) - P_{r} I(t)}, \\ \frac{dI}{dt} = \frac{(1-P_{r}) q_{0} \beta S(t) I(t)}{N(t) - P_{r} I(t)}- \gamma I, \\ \frac{dR}{dt} = \gamma I, \end{gathered} $$

with the initial conditions $S(0) = S_{0}$, $I(0) = I_{0}$ and $R(0) = 0$. The analysis shows that the proportion $z_{\infty}$ of individuals that experience the infection at the end of the epidemic is the solution of the non-linear algebraic equation

8 $$ z_{\infty}= \bigl( (1 - P_{r})^{2} R_{0} + P_{r} \bigr) \bigl( (1-z_{\infty})^{-\frac{P_{r}}{ {R}_{0}( 1 - P_{r})} } - 1 \bigr) / \bigl( R_{0} P_{r} (1-P_{r}) \bigr), $$

where $P_{r} \in[0, 1]$ and ${R}_{0} = q_{0} \beta/\gamma$ is the basic reproduction number for model (7) if the reduction $P_{r} = 0$. Equation (8) could be reformulated as

9 $$\begin{aligned} z_{\infty} =& 1 - { \biggl(\frac{P_{r} + (1-P_{r})^{2} R_{0}}{P_{r} + (1-P_{r})^{2} R_{0} + P_{r}(1-P_{r})R_{0} z_{\infty}} \biggr)}^{{\frac{(1-P_{r})R_{0}}{P_{r}}} } \\ = & 1 - { \biggl(1 + \frac{P_{r}(1-P_{r})R_{0} z_{\infty}}{P_{r} + (1-P_{r})^{2} R_{0}} \biggr)}^{{-\frac{(1-P_{r})R_{0}}{P_{r}}} }. \end{aligned}$$

It is easy to check that, in the limiting case $P_{r} \to0$, Eq. (9) is reduced to the popular and well-known form

10 $$ \bar{z}_{\infty}= 1 - \exp ({-{R}_{0} \bar{z}_{\infty}} ), $$

where $\bar{z}_{\infty}= \lim_{{P}_{r}\to0}{z_{\infty}}$.

### Rescaled endemic model

On putting $x = S/N$, $y = I/N$ and $z = R/N$, we get

11 $$\begin{aligned}& \frac{dx}{dt} = \mu- \frac{(1-P_{r}) q_{0} \beta x y}{1 - P_{r} y} - \mu x, \\& \frac{dy}{dt} = \frac{(1-P_{r}) q_{0} \beta x y}{1 - P_{r} y} - (\gamma + \mu) y, \\& \frac{dz}{dt} = \gamma y - \mu z, \end{aligned}$$

where

$$x + y + z = 1. $$

**Figure 1:**
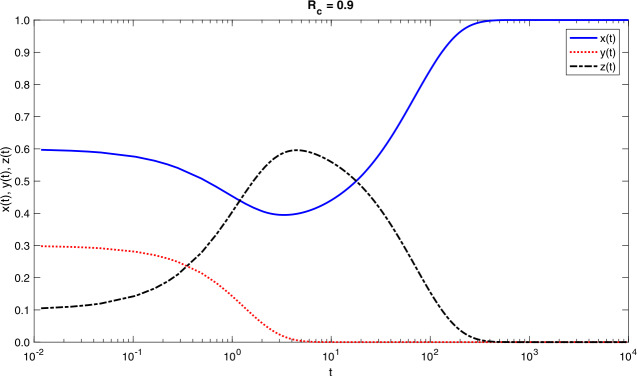
Time-dependent solutions for model (11) for $\mathcal{R}_{c} = 0.9$ and initial conditions $x(0) = 0.6$, $y(0) = 0.3$ and $z(0) = 0.1$. Model parameter values are as shown in Table 1.

**Figure 2:**
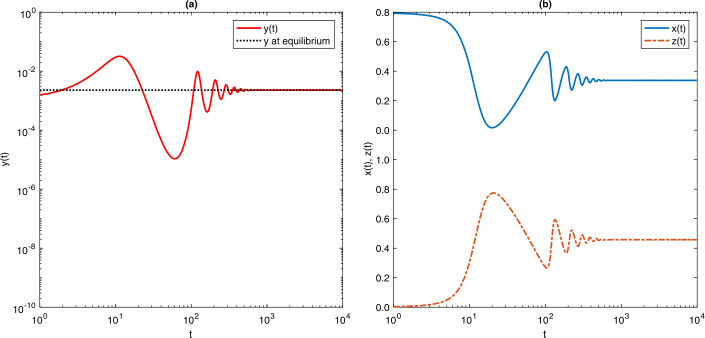
Time-dependent solutions for model (11) for $\mathcal{R}_{c} = 1.3$ and initial conditions $x(0) = 0.999$, $y(0) = 0.001$ and $z(0) = 0.0$. The prevalence of infection at equilibrium is $\bar{y} = 0.0023$. Model parameter values are as shown in Table 1.

If $P_{r} = 0$ and $q_{0} = 1$, the model is connected to the simple SIR model with proportions and standard incidence “*force of infection*”. The time-dependent solutions of model (11) are shown in Figs. 1 and 2.

**Table 1:** Physical meaning, value, dimension and references for model states and parameters. (Dim. = Dimension, Ref. = References, Est. = Estimated, Arbit. = Arbitrary)

Symbol	Description	Value	Dim.	Ref.
*S*(*t*)	Total number of susceptible individuals at time *t*.	–	–	–
*I*(*t*)	Total number of infected individuals at time *t*.	–	–	–
*R*(*t*)	Total number of recovered individuals at time *t*.	–	–	–
*μ*	Per-capita birth/death rate.	1/70	Year^−1^	[8, 20]
*β*	Per-capita effective contact rate at which susceptible individuals acquire the infection.	553.77	Year^−1^	Est.
*γ*	Per-capita recovery rate for infected individuals.	365/3.38	Year^−1^	[20, 27]
*α*	Per-capita treatment rate for infected individuals.	365/2	Year^−1^	Arbit.
$R_{\Diamond}$	The basic reproduction number for model (11).	1.525	–	[20, 27]
$p_{0}$	The proportion of time during which a non-infected individual stays at rest.	0.2	–	Arbit.
$p_{1}$	The proportion of time during which an infected individual stays at rest.	0.3	–	Arbit.
$q_{0}$	The proportion of time during which a non-infected individual mingles with other individuals in the total population.	0.8	–	Arbit.
$q_{1}$	The proportion of time during which an infected individual mingles with other individuals in the total population.	0.7	–	Arbit.
$\widehat{P}_{r}$	The percent reduction in the mingling proportion of time due to acquiring the infection.	12.5	–	Arbit.

### Equilibrium analysis for the rescaled endemic model (11)

The equilibrium analysis of model (11) shows that it has the infection-free equilibrium (IFE) $E_{0} = (1, 0, 0)^{\prime}$, where the prime ′ denotes vector transpose. This trivial equilibrium is locally asymptotically stable if and only if the control reproduction number $\mathcal{R}_{c} < 1$, where

12 $$ \mathcal{R}_{c} = \frac{(1-P_{r}) q_{0} \beta}{\gamma+ \mu} = (1-P_{r}) \mathcal{R}_{\Diamond}, $$

where

13 $$ \mathcal{R}_{\Diamond} = \frac{q_{0} \beta}{\gamma+ \mu} $$

is the basic reproduction number for model (11) if the there is no behavioral change between infected and non-infected individuals (i.e., $P_{r} = 0$). The analysis shows further that model (11) has a unique endemic equilibrium (UEE) $E = (\bar{x}, \bar{y}, \bar{z})^{\prime}$, where

14 $$\begin{aligned}& \bar{x} = \frac{ \gamma+ (1-P_{r}) \mu}{(1-P_{r}) (\gamma+ \mu) \mathcal{R}_{\Diamond} - P_{r} \mu} = \biggl(1 - P_{r} \frac{D_{I}}{L_{0}} \biggr) \Big/ \biggl( {(1 - P_{r}) \mathcal{R}_{\Diamond} - P_{r} \frac {D_{I}}{L_{0}}} \biggr), \end{aligned}$$

15 $$\begin{aligned}& \begin{aligned}[b]\bar{y} & = \frac{\mu[(1-P_{r}) q_{0} \beta- (\gamma+\mu)]}{(\gamma+\mu )[(1-P_{r}) q_{0} \beta- P_{r} \mu]} = \frac{D_{I}(\mathcal{R}_{c} - 1)}{L_{0} \mathcal{R}_{c} - P_{r} D_{I}} \\ &= \frac{D_{I}}{L_{0}} \bigl( {(1-P_{r}) \mathcal{R}_{\Diamond} - 1} \bigr)\Big/ \biggl({(1-P_{r}) \mathcal{R}_{\Diamond} - P_{r} \frac {D_{I}}{L_{0}}} \biggr) , \end{aligned} \end{aligned}$$

16 $$\begin{aligned}& \bar{z} = \frac{\gamma}{\mu} \bar{y} = \biggl( 1 - \frac {D_{I}}{L_{0}} \biggr) \bigl( {(1-P_{r}) \mathcal{R}_{\Diamond} - 1} \bigr) \Big/ \biggl({(1-P_{r}) \mathcal{R}_{\Diamond} - P_{r} \frac {D_{I}}{L_{0}}} \biggr) , \end{aligned}$$

and

- $D_{I} = 1/(\gamma+\mu)$ is the duration of time spent in the infected state,
- $L_{0} = 1/\mu$ is the expected time of life at birth,
- $D_{I} / L_{0}$ is the proportion of life-time during which an individual is infected.

#### Proposition 2

*Model* (11) *has*

- *an IFE*$E_{0} = (1, 0, 0)^{\prime}$*which is locally asymptotically stable if and only if*$\mathcal{R}_{c} \le1$*and is unstable if*$\mathcal {R}_{c} > 1$;
- *an endemic equilibrium* (*EE*) $E = (\bar{x}, \bar{y}, \bar {z})^{\prime}$*that exists if and only if*$\mathcal{R}_{c} > 1$.

It is noteworthy that the UEE *E* exists if and only if the control reproduction number $\mathcal{R}_{c} > 1$. Moreover, one can check that

17 $$ \frac{\partial\bar{y}}{\partial\mathcal{R}_{c}} = \frac{D_{I}}{L_{0}} \biggl(1 - P_{r} \frac{D_{I}}{L_{0}} \biggr)\Big/ { \biggl( \mathcal{R}_{c} - P_{r} \frac {D_{I}}{L_{0}} \biggr)}^{2}> 0. $$

The relation (17) says that the proportion of infected individuals *ȳ* in the endemic situation increases with the increase of the control reproduction number $\mathcal{R}_{c}$. Moreover $\bar{y} = 0$ at $\mathcal{R}_{c} = 1$, while

18 $$ \lim_{\mathcal{R}_{c}\rightarrow\infty}{\bar{y}} = \frac{D_{I}}{L_{0}} < 1. $$

Hence, we show the following proposition.

#### Proposition 3

*The endemic prevalence of infectionȳincreases monotonically with the increase of the control reproduction number*$\mathcal{R}_{c}$*and has a supremum*$\sup{\bar{y}} = D_{I} / L_{0}$.

On the other hand, Eq. (14) says that the proportion *x̄* of susceptible individuals in the endemic situation does not equal the inverse $1/\mathcal{R}_{c}$ of the control reproduction number. However, this property holds if and only if $P_{r} = 0$. The following proposition summarizes the above result.

#### Proposition 4

*The proportion of susceptible individuals in the endemic situation does not equal the inverse of the effective reproduction number* $\mathcal {R}_{c}$*but it is given by*

$$ \bar{x} = \biggl(1 - P_{r} \frac{D_{I}}{L_{0}} \biggr) \Big/ \biggl( { \mathcal {R}_{c} - P_{r} \frac{D_{I}}{L_{0}}} \biggr). $$

### Cross-sectional analysis

If a cross-sectional survey has been applied to determine the proportions *x̄*, *ȳ* and *z̄* of subpopulations in the endemic situation, then we may solve the two equations (15) and (16) together to get

19 $$ \frac{D_{I}}{L_{0}} = \frac{\bar{y}}{\bar{y} + \bar{z}}= \frac{\bar{y}}{1 - \bar{x}}. $$

Now, we use (19) in (14) and solve in terms of $\mathcal{R}_{\Diamond} $ to get

$$ \mathcal{R}_{\Diamond} = \frac{1 - P_{r} \bar{y}}{(1 - P_{r}) \bar{x}}. $$

Hence,

20 $$ \mathcal{R}_{c} = (1-P_{r}) \mathcal{R}_{\Diamond} = \frac{1 - P_{r} \bar {y}}{\bar{x}} < \frac{1}{\bar{x}}. $$

Equation (19) says that, if the life expectancy $L_{0}$ is known, then a cross-sectional study may help estimate the average length of the infectious period. However, Eq. (20) says that $\mathcal{R}_{c}$ could be estimated from a cross-sectional study once *x̄*, *ȳ* and $P_{r}$ are known. It says also that the control reproduction number $\mathcal{R}_{c}$ decreases with the increase of $P_{r}$. Moreover, since $\mathcal{R}_{\Diamond} = \mathcal{R}_{c}\vert _{P_{r} = 0}$, then the percent reduction in $\mathcal{R}_{c}$ due to a $\widehat{P}_{r}$ percent reduction in infected individuals time activity is $\widehat{\mathcal{R}}_{c}$ where

21 $$\begin{aligned} \widehat{\mathcal{R}}_{c} = \frac{\mathcal{R}_{\Diamond} - \mathcal {R}_{c}}{\mathcal{R}_{\Diamond}} = P_{r} = \frac{\widehat{P}_{r} }{100} \end{aligned}$$

and $\widehat{P}_{r} = (100 \times P_{r})$.

### Stability analysis

To establish the local stability analysis of the EE $E = (\bar{x}, \bar {y}, \bar{z})^{\prime}$, we linearize model (11) around that equilibrium and compute the corresponding $3 \times3$ Jacobian matrix *J*. This matrix has an eigenvalue −*μ*, while its other two eigenvalues are those of the submatrix

22 $$ J_{\mathrm{sub}} = \left ( \textstyle\begin{array}{c@{\quad}c} -{\mu}/{\bar{x}} & - (\gamma+\mu) \bar{y} / (\bar{x} \mathcal{R}_{c})\\ \mu(1-\bar{x})/\bar{x} & -(\gamma+ \mu)[1 + \bar{y} / (\bar{x} \mathcal{R}_{c})] \end{array}\displaystyle \right ). $$

It is easy to check that the determinant of $J_{\mathrm{sub}}$ is

$$ \det({J_{\mathrm{sub}}}) = \frac{\mu(\gamma+\mu)}{\bar{x}} \biggl(1 - \frac{\bar {y}}{\mathcal{R}_{c}} \biggr) > \frac{\mu(\gamma+\mu)}{\bar{x}} \biggl(1 - \frac{1}{\mathcal{R}_{c}} \biggr) , $$

which is positive for values of $\mathcal{R}_{c} > 1$. It can also be checked that its trace reads

$$ \operatorname{Tr}(J_{\mathrm{sub}}) = -(\gamma+ \mu) - \frac{\mu}{\bar{x}} \biggl( 1 - \frac{L_{0}}{D_{I}} \cdot\frac{\bar{y}}{\mathcal{R}_{c}} \biggr). $$

From (18), we have $\bar{y} < D_{I}/L_{0}$, which implies that $L_{0} \bar{y} / D_{I} < 1$. It induces, for $\mathcal{R}_{c} > 1$, that

$$ \frac{L_{0}}{D_{I}} \cdot\frac{\bar{y}}{\mathcal{R}_{c}} < 1. $$

Hence, the trace of the matrix $J_{\mathrm{sub}}$ is negative, for values of $\mathcal{R}_{c} > 1$. Therefore, the eigenvalues of the Jacobian matrix *J* are all negative for $\mathcal{R}_{c} > 1$ and we show the following proposition.

#### Proposition 5

*The EE*$E=(\bar{x}, \bar{y}, \bar{z})^{\prime}$*is locally asymptotically stable if and only if*$\mathcal{R}_{c} > 1$.

Based on the above analysis, the infection does not persist if the control reproduction number $\mathcal{R}_{c} < 1$. This is equivalent to having

23 $$ P_{r} > 1 - \frac{1}{\mathcal{R}_{\Diamond}}. $$

The condition (23) says that the infection could be eliminated from the population if control measures aiming at exceeding the percent reduction in the mingling time of infected individuals compared to non-infected ones to slightly above $100 (1 - 1/\mathcal {R}_{\Diamond})$. Other control strategies may include vaccination of newborns or treating infected individuals, which are going to be discussed below.

## Herd immunity

If we assume that a proportion *p* of newborns gets vaccinated immediately after birth, then the resulting rescaled model reads

24 $$ \begin{gathered} \frac{dx}{dt} = (1-p) \mu- \frac{(1-P_{r}) q_{0} \beta x y}{1 - P_{r} y} - \mu x, \\ \frac{dy}{dt} = \frac{(1-P_{r}) q_{0} \beta x y}{1 - P_{r} y} - (\gamma + \mu) y, \\ \frac{dz}{dt} = p \mu+ \gamma y - \mu z. \end{gathered}$$

Model (24) has the IFE $E_{0v} = (1-p , 0 , p)^{\prime}$ in addition to the UEE $E_{v} = (\bar{x}_{v}, \bar{y}_{v}, \bar{z}_{v})^{\prime}$, where

$$\begin{aligned}& \bar{x}_{v} = \biggl(1 - (1-p) P_{r} \frac{D_{I}}{L_{0}} \biggr) \Big/ \biggl( {(1 - P_{r}) \mathcal{R}_{\Diamond} - P_{r} \frac{D_{I}}{L_{0}}} \biggr) , \\& \bar{y}_{v} = \frac{D_{I}}{L_{0}} \bigl( {(1-p) (1-P_{r}) \mathcal {R}_{\Diamond} - 1} \bigr) \Big/ \biggl({(1-P_{r}) \mathcal{R}_{\Diamond} - P_{r} \frac{D_{I}}{L_{0}}} \biggr) , \\& \bar{z}_{v} = p + \biggl( 1 - \frac{D_{I}}{L_{0}} \biggr) \bigl( {(1-p) (1-P_{r}) \mathcal{R}_{\Diamond} - 1} \bigr)\Big/ \biggl({(1-P_{r}) \mathcal{R}_{\Diamond} - P_{r} \frac{D_{I}}{L_{0}}} \biggr). \end{aligned}$$

This UEE exists if and only if the effective reproduction number in the presence of vaccination

25 $$ \mathcal{R}_{v} = (1 -p){\mathcal{R}_{c}} = (1-p) (1-P_{r}) \mathcal {R}_{\Diamond}$$

is bigger than one. In a similar way to the previous section, $E_{0v}$ could be proven locally asymptotically stable if and only if $\mathcal {R}_{v} < 1$, while $E_{v}$ could be shown to be locally asymptotically stable whenever it exists. Therefore, we show the following proposition.

### Proposition 6

*If a proportionpof newborns is vaccinated immediately after birth*, *then the resulting model* (24) *has an IFE that is locally asymptotically stable if and only if*$\mathcal{R}_{v} < 1$. *Moreover*, *model* (24) *has a UEE that exists and is locally asymptotically stable if and only if*$\mathcal{R}_{v} > 1$.

The inequality $\mathcal{R}_{v} < 1$ is equivalent to $p > p_{c}$, where

26 $$ p_{c} = 1 - \frac{1}{(1-P_{r}) \mathcal{R}_{\Diamond}} = 1 - \frac{100}{(100 - \widehat{P}_{r}) \mathcal{R}_{\Diamond}} $$

is the critical vaccination coverage level required to eliminate the infection. It is clear that $p_{c} > 0$ if and only if $P_{r} > 1 - 1/\mathcal{R}_{\Diamond}$. Also, it is noteworthy that $p_{0} = (1 - 1/{\mathcal{R}_{\Diamond}}) \ge p_{c}$ is the critical vaccination coverage level required to eliminate the infection if infected individuals do not reduce their contact-activity time (i.e., $\widehat{P}_{r} = 0$). Therefore,

$$ p_{0} - p_{c} = \frac{\widehat{P}_{r}}{(100 - \widehat{P}_{r}) \mathcal {R}_{\Diamond}} $$

represents the reduction in that critical vaccination coverage level due to a $\widehat{P}_{r} = (100 \times P_{r})$ percent reduction in the contact time activity of infected individuals. Thus, the percent reduction in that critical vaccination coverage level due to a $\widehat {P}_{r} $ percent reduction in the contact time activity of infected individuals is $\widehat{\mathcal{P}_{c}} = 100 \times \widehat{p}_{c}$, where

27 $$ \widehat{p}_{c} = \frac{p_{0} - p_{c}}{p_{0}} = \frac{\widehat{P}_{r}}{(100 - \widehat{P}_{r})(\mathcal{R}_{\Diamond} - 1)}. $$

Therefore,

28 $$ \widehat{\mathcal{P}_{c}} = \frac{100 \times \widehat{P}_{r}}{(100 - \widehat{P}_{r}) (\mathcal{R}_{\Diamond}- 1)}. $$

**Figure 3:**
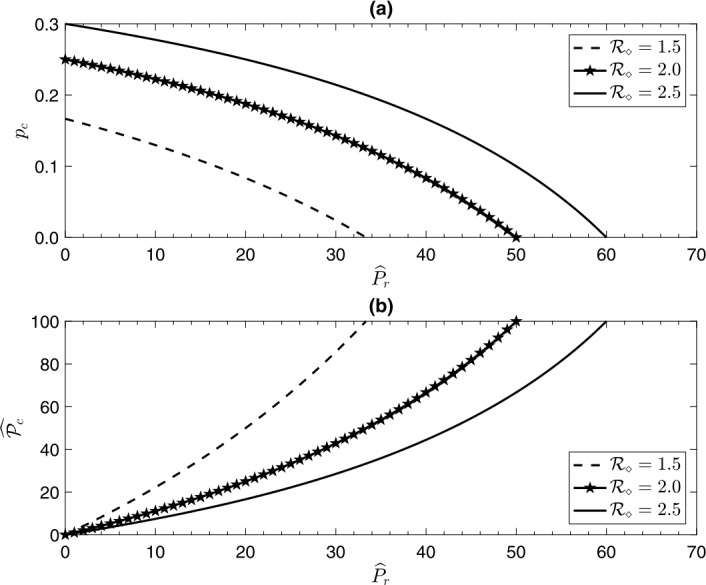
The critical vaccination coverage level $p_{c}$ required to eliminate the infection as a function of the percent reduction in the contact time activity of infected individuals $\widehat{P}_{r}$ is shown in part (**a**), while the percent reduction in the critical vaccination coverage level $\widehat{\mathcal{P}_{c}}$ required to eliminate the infection as a function of the percent reduction in the contact time activity of infected individuals $\widehat{P}_{r}$ is shown in part (**b**), for different values of the basic reproduction number $\mathcal{R}_{\Diamond}$

It is clear that $\widehat{\mathcal{P}_{c}} = 100$ when $\widehat{P}_{r} = 100 ( 1 - 1 / \mathcal{R}_{\Diamond})$. Moreover, it is easy to check that $\partial{\widehat{\mathcal{P}_{c}}} / \partial{\mathcal {R}_{\Diamond}} < 0$, while $\partial{\widehat{\mathcal{P}_{c}}}/\partial {\widehat{P}_{r}} > 0$. Hence, the higher the percent reduction in infected individuals time activity is, the higher the percent reduction in the critical vaccination coverage level is. Figure 3 shows both $p_{c}$ and $\widehat{\mathcal{P}_{c}}$ as functions of $\widehat {P}_{r}$, for three different values of the basic reproduction number $\mathcal{R}_{\Diamond}$. Part (a) of the figure shows that $p_{c}$ decreases with the increase of $\widehat{P}_{r}$ and increases with the increase of $\mathcal{R}_{\Diamond}$. Thus, the higher the percent reduction in infected individuals contact-activity time is, the lower the critical vaccination coverage required to eliminate the infection is. On the other hand, part (b) of Fig. 3 shows that $\widehat{\mathcal{P}_{c}}$ increases with the increase of $\widehat {P}_{r}$ and decreases with the increase of $\mathcal{R}_{\Diamond}$. In epidemiological terms, we deduce that the higher the percent reduction in infected individuals’ contact-activity time is, the higher the percent reduction in the critical vaccination coverage required to eliminate the infection is which in turn reduces the cost of vaccination needed to protect the population from the infection.

Equation (28) may help us determine the required reduction in infected individuals contact-activity time if vaccination coverage is limited. For example, the value of $\widehat{P}_{r}$ corresponding to a 50% reduction in the critical vaccination coverage is given by

29 $$ \widehat{P}_{r}^{50\%} = \frac{100 (\mathcal{R}_{\Diamond}- 1)}{\mathcal {R}_{\Diamond}+ 1} = 100 \biggl( 1 - \frac{2}{\mathcal{R}_{\Diamond}+ 1} \biggr) , $$

which increases with the increase of the basic reproduction number $\mathcal{R}_{\Diamond}$. Consequently, we show the following proposition.

### Proposition 7

*The percent reduction in the critical vaccination coverage level required to eliminate the infection due to a*$\widehat{P}_{r}$*percent reduction in the contact*-*activity time of infected individuals is given by Eq*. (28). *Moreover*, *a* 50% *reduction in the cost of eliminating the infection is attained by reducing the infected individuals’ contact*-*activity time by*$100 {(\mathcal{R}_{\Diamond} - 1 )} / (\mathcal{R}_{\Diamond} + 1 )$*percent*.

## Maximum capacity treatment

Assume that the infection is treatable and there is a maximum treatment capacity, in the sense that at most a proportion $\mathcal{Y} = \mathcal {I}/N > 0$ could be treated with rate *α*. Hence, for $I < \mathcal {I}$, the total number of infected individuals who get treated at time *t* is *αI*, while if $I \ge\mathcal{I}$ it is $\alpha\mathcal {I}$. In mathematical terms, the total number of treated individuals at time *t* is in stepwise form and reads

30 $$ T(I) = \left \{ \textstyle\begin{array}{l@{\quad}l} \alpha I & \text{if } I < \mathcal{I}, \\ \alpha\mathcal{I} & \text{if } I \ge\mathcal{I}. \end{array}\displaystyle \right . $$

Hence, model (11) with treatment reads

31 $$\begin{aligned}& \frac{dx}{dt} = \mu- \frac{(1-P_{r}) q_{0} \beta x y}{1 - P_{r} y} - \mu x, \\& \frac{dy}{dt} = \frac{(1-P_{r}) q_{0} \beta x y}{1 - P_{r} y} - (\gamma + \mu) y - T(y), \\& \frac{dz}{dt} = \gamma y + T(y) - \mu z, \end{aligned}$$

where

32 $$ T(y) = \left \{ \textstyle\begin{array}{l@{\quad}l} \alpha y & \text{if } y < \mathcal{Y}, \\ \alpha\mathcal{Y} & \text{if } y \ge\mathcal{Y}, \end{array}\displaystyle \right . $$

represents the proportion of infected individuals who get treated per unit time.

### Equilibrium and stability analyses for model (31)

The equilibrium analysis shows that model (31) has the IFE $E_{0, T} = (1, 0, 0)^{\prime}$ which could easily be proven to be locally asymptotically stable if and only if the effective reproduction number in the presence of treatment $\mathcal{R}_{T} < 1$, where

33 $$ \mathcal{R}_{T} = \frac{(1-P_{r}) q_{0} \beta}{\gamma+ \mu+ \alpha} = (1-P_{r}) \mathcal{R}_{\Diamond}^{T}, $$

where $\mathcal{R}_{\Diamond}^{T} = q_{0} \beta/(\gamma+ \mu+ \alpha)$ is the basic reproduction number of model (31) in the absence of any behavioral change for infected individuals (i.e., $P_{r} = 0$). Moreover, the analysis shows that model (31) has endemic equilibria for which two cases arise.

*Case (1)*: $I < \mathcal{I} $ (i.e., $y < \mathcal{Y}$).
  In this case, there is a bifurcation point in the plane $(\beta, y)$ at the point $\mathcal{P}_{0} = (\beta_{0}, 0)$, where
  34 $$ \beta_{0} = \frac{\gamma+ \mu+ \alpha}{q_{0}(1-P_{r})}, $$
  at which the bifurcation is forward (i.e., supercritical). The EE is $E_{1} = (x_{1}, y_{1}, z_{1})^{\prime}$, where
  35 $$\begin{aligned}& x_{1} = \frac{\gamma+ \mu+ \alpha- P_{r} \mu}{(1-P_{r}) q_{0} \beta- P_{r} \mu} = \frac{1 - P_{r} D_{I}^{T}/L_{0}}{(1-P_{r}) \mathcal{R}_{\Diamond}^{T} - P_{r} D_{I}^{T} / L_{0}} = \frac{1 - P_{r} D_{I}^{T}/L_{0}}{\mathcal{R}_{T} - P_{r} D_{I}^{T} / L_{0}}, \\ & \begin{aligned}[b]y_{1} & = \frac{\mu}{\gamma+\mu+ \alpha} \times \frac{ (1-P_{r}) q_{0} \beta- (\gamma+ \mu+ \alpha)}{ (1-P_{r}) q_{0} \beta- P_{r} \mu}, \\ & = \frac{D_{I}^{T}}{L_{0}} \times \frac{ (1-P_{r}) \mathcal{R}_{\Diamond }^{T} - 1}{ (1-P_{r}) \mathcal{R}_{\Diamond}^{T} - P_{r} D_{I}^{T} / L_{0}} = \frac{D_{I}^{T}}{L_{0}} \times \frac{\mathcal{R}_{T} - 1}{\mathcal{R}_{T} - P_{r} D_{I}^{T} / L_{0}}, \end{aligned} \\ & \begin{aligned}z_{1} &= \biggl( 1 - \frac{\mu}{\gamma+ \mu+ \alpha} \biggr) \frac {(1-P_{r}) q_{0} \beta- (\gamma+ \mu+ \alpha)}{(1-P_{r}) q_{0} \beta- P_{r} \mu} \\ & = \biggl(1 - \frac{D_{I}^{T}}{L_{0}} \biggr) \frac{ (1-P_{r}) \mathcal {R}_{\Diamond}^{T} - 1}{ (1-P_{r}) \mathcal{R}_{\Diamond}^{T} - P_{r} D_{I}^{T} / L_{0}} = \biggl( 1 - \frac{D_{I}^{T}}{L_{0}} \biggr) \frac{\mathcal{R}_{T} - 1}{\mathcal{R}_{T} - P_{r} D_{I}^{T} / L_{0}},\end{aligned} \end{aligned}$$
  and $D_{I}^{T} = 1/(\gamma+\mu+ \alpha)$ is the time spent in the infected state in the presence of treatment.
  It is noteworthy that the EE $E_{1}$ does exist if and only if
  36 $$ \frac{1}{1 - P_{r}} < \mathcal{R}_{\Diamond}^{T} < \frac{(1 - P_{r} \mathcal{Y}) D_{I}^{T} / L_{0}}{(1-P_{r}) (D_{I}^{T} / L_{0} - \mathcal {Y})}. $$

The following proposition summarizes the above results.

#### Proposition 8

*If*$y < \mathcal{Y}$, *then model* (31) *has*

- *an IFE*$E_{0, T} = (1, 0, 0)^{\prime}$*which is locally asymptotically stable if and only if*$\mathcal{R}_{T} < 1$;
- *a bifurcation point*$\mathcal{P}_{0} = (\beta_{0}, 0)$*in the plane*$(\beta, y)$*at which the bifurcation is supercritical* (*forward*);
- *a UEE that exists if and only if the inequality* (36) *holds*.

The stability analysis shows that the EE $E_{1}$ is locally asymptotically stable whenever it exists and, therefore, we show the following proposition whose proof is deferred to Appendix B.

#### Proposition 9

*The EE*$E_{1}$*for model* (31) *is locally asymptotically stable whenever it exists*.

*Case (2)*: $I \ge\mathcal{I} $ (i.e., $y \ge\mathcal{Y}$).
  In this case, the mathematical computations show that the proportions of the three subpopulations in the endemic situation satisfy the relations
  37 $$\begin{aligned}& \widetilde{x} = \biggl( 1 - \frac{\alpha}{\mu} \mathcal{Y} \biggr) - \frac{\gamma+ \mu}{\mu} \widetilde{y}, \end{aligned}$$
  38 $$\begin{aligned}& \widetilde{z} = \frac{\gamma}{\mu} \widetilde{y} + \frac{\alpha}{\mu } \mathcal{Y} , \end{aligned}$$
  and *ỹ* is the solution of the quadratic equation
  39 $$ \begin{aligned}[b] F(\beta, \widetilde{y}) & = \bigl( (1-P_{r}) q_{0} \beta- P_{r} \mu \bigr) ( \gamma+ \mu ) {\widetilde{y}}^{2} \\ &\quad+ \bigl( \mu (\gamma+ \mu) - P_{r} \mu \alpha \mathcal{Y} - (1-P_{r}) (\mu- \alpha\mathcal{Y}) q_{0} \beta \bigr) \widetilde{y} + \mu \alpha \mathcal{Y}\\ & = 0,\end{aligned} $$
  where $\mathcal{Y} \le\widetilde{y} < 1$. Once a solution *ỹ* of (39) is obtained, we substitute in (37) and (38) to get the other two proportions, *x̃* and *z̃*. Therefore, there is a one-to-one correspondence between the solution(s) of (39) and those of (37) and (38).
  Now, it is easy to check from (35) that
  $$ \frac{\partial y_{1}}{\partial\mathcal{R}_{\Diamond}^{T}} = \frac {D_{I}^{T}}{L_{0}} \times\frac{(1-P_{r}) (1 - P_{r} D_{I}^{T}/L_{0} )}{ ( (1-P_{r}) \mathcal{R}_{\Diamond}^{T} - P_{r} D_{I}^{T}/L_{0} )^{2}} > 0, $$
  which means that $y_{1}$ increases with the increase of $\mathcal {R}_{\Diamond}^{T}$. Moreover, $y_{1}$ reaches its maximum $\mathcal{Y}$ at
  $$ \mathcal{R}_{\Diamond}^{T} = \frac{(1 - P_{r} \mathcal{Y}) D_{I}^{T} / L_{0}}{(1-P_{r}) (D_{I}^{T} / L_{0} - \mathcal{Y})}, $$
  which is equivalent to having $\beta= \beta_{1}$, where
  40 $$ \beta_{1} = \frac{1 - P_{r} \mathcal{Y}}{q_{0} (1-P_{r}) (D_{I}^{T} - \mathcal{Y} L_{0})} = \frac{(1-P_{r} \mathcal{Y}) \mu (\gamma+ \mu+ \alpha)}{q_{0} (1-P_{r}) ( \mu- (\gamma+ \mu+ \alpha)\mathcal{Y} )}. $$
  On the other hand, if we put $\widetilde{y} = \mathcal{Y}$ in (39) and solve with respect to *β*, we get $\beta = \beta_{1}$. Hence, Eq. (39) could be seen as a bifurcation equation and the point $\mathcal{P}_{1} = (\beta_{1}, \mathcal {Y})$ is a bifurcation point in the plane $(\beta, y)$ for $y \in [\mathcal{Y}, 1]$. It is worth noting that the bifurcation point $\mathcal{P}_{1}$ does exist only if
  41 $$ \mathcal{Y} < \frac{\mu}{\gamma+ \mu+ \alpha}:= \mathcal{Y}_{1}. $$

Therefore, we show the following proposition.

#### Proposition 10

*If*$I \ge\mathcal{I}$ (*i*.*e*., $y \ge\mathcal{Y}$), *then model* (31) *has a bifurcation point*$\mathcal{P}_{1} = (\beta_{1}, \mathcal {Y})$*in the plane*$(\beta, \widetilde{y})$, *which exists if and only if*$\mathcal{Y} < \mu / (\gamma+ \mu+ \alpha)$.

At the point $\mathcal{P}_{1}$ we compute the direction of bifurcation. To this end, we make use of the implicit function theorem [22, 25]. The mathematical computations show that

$$ \frac{\partial F}{\partial\beta}\bigg\vert _{(\beta_{1}, \mathcal{Y})} = - q_{0} (1 - P_{r}) \mathcal{Y} \bigl( \mu- (\gamma+ \mu+ \alpha) \mathcal {Y} \bigr) $$

and

$$\begin{aligned} \frac{\partial F}{\partial\widetilde{y}}\bigg\vert _{(\beta_{1}, \mathcal {Y})} =& (1-P_{r}) q_{0} \beta_{1} \bigl( (\gamma+ \mu+ \alpha) \mathcal{Y} - \mu \bigr) + (1-P_{r}) q_{0} \beta_{1} (\gamma+ \mu) \mathcal{Y} \\ &{} - P_{r} \mu\mathcal{Y} (\gamma+ \mu+ \alpha) - P_{r} \mu\mathcal {Y} (\gamma+ \mu) + \mu(\gamma+ \mu). \end{aligned}$$

On making use of (40) and simplifying, we get

$$\begin{aligned} \frac{\partial F}{\partial\widetilde{y}}\bigg\vert _{(\beta_{1}, \mathcal {Y})} =& (1-P_{r}) q_{0} \beta_{1} \mathcal{Y}(\gamma+ \mu) - \mu\alpha- P_{r} \mu\mathcal{Y} (\gamma+ \mu) \\ = & \frac{\mu(\gamma+ \mu)(\gamma+ \mu+ \alpha) \mathcal{Y} (1 - P_{r} \mathcal{Y})}{\mu- (\gamma+ \mu+ \alpha) \mathcal{Y}} - \bigl(\mu \alpha+ P_{r} \mu\mathcal{Y} (\gamma+ \mu) \bigr) \\ = & \frac{\mu(\gamma+ \mu) \mathcal{Y} (\gamma+ \alpha+ (1-P_{r})\mu ) - \mu\alpha ( \mu- (\gamma+ \mu+ \alpha )\mathcal{Y} )}{\mu- (\gamma+ \mu+ \alpha) \mathcal{Y}}. \end{aligned}$$

Thus,

42 $$\begin{aligned} \frac{d \widetilde{y}}{d\beta}\bigg\vert _{(\beta_{1}, \mathcal{Y})} =& - \frac{\partial F}{\partial\beta} \bigg\vert _{(\beta_{1}, \mathcal{Y})}\Big/ \frac{\partial F}{\partial\widetilde{y}}\bigg\vert _{(\beta_{1}, \mathcal{Y})} \\ = & \frac{ q_{0} (1-P_{r}) \mathcal{Y} { ( \mu- (\gamma+ \mu+ \alpha )\mathcal{Y} )}^{2} }{\mu ( \mathcal{Y} (\gamma+ \mu) (\gamma+ \alpha+ (1-P_{r}) \mu) + \alpha(\gamma+ \mu+ \alpha)\mathcal{Y} - \alpha\mu )}. \end{aligned}$$

Thus, the direction of bifurcation depends on the sign of the denominator of (42). Accordingly, at the bifurcation point $\mathcal{P}_{1}$, the bifurcation is backward (subcritical) if and only if

$$ \bigl((\gamma+ \mu) \bigl(\gamma+ \alpha+ (1-P_{r})\mu\bigr) + \alpha(\gamma+ \alpha+ \mu) \bigr) \mathcal{Y} - \alpha\mu< 0, $$

which is equivalent to

43 $$ \mathcal{Y} < \frac{\alpha\mu}{(\gamma+ \mu)(\gamma+ \alpha+ (1-P_{r})\mu) + \alpha(\gamma+ \alpha+ \mu)} := \mathcal{Y}_{2}. $$

Consequently, we state the following proposition.

#### Proposition 11

*If*$I \ge\mathcal{I}$ (*i*.*e*., $y \ge\mathcal{Y}$), *then model* (31) *exhibits backward bifurcation at the point*$\mathcal{P}_{1} = (\beta_{1}, \mathcal{Y})$*if and only if the inequality* (43) *holds*.

The condition (43) is necessary to the existence of two feasible solutions to (39). The two solutions are given by

44 $$ {y}_{2} = \frac{- B - \sqrt{B^{2} - 4 A C}}{2 A}, \qquad {y}_{3} = \frac{- B + \sqrt{B^{2} - 4 A C}}{2 A} , $$

where

45 $$\begin{aligned}& A = \bigl( (1-P_{r}) q_{0} \beta- P_{r} \mu \bigr) ( \gamma+ \mu ), \\& B = \bigl( \mu (\gamma+ \mu) - P_{r} \mu \alpha \mathcal{Y} - (1-P_{r}) (\mu- \alpha\mathcal{Y}) q_{0} \beta \bigr), \\& C = \mu \alpha \mathcal{Y}. \end{aligned}$$

These two solutions collide and mutually annihilate at $\beta= \beta _{2}$ (see Appendix C for a complete derivation of Eq. (46)), where

46 $$ \beta_{2} = \frac{\mu{ ( \sqrt{\mu(\gamma+\mu)} + \sqrt{\alpha \mathcal{Y} ( \gamma+ \mu- P_{r}(\mu- \alpha\mathcal{Y}) )} )}^{2}}{q_{0} (1-P_{r}) (\mu- \alpha\mathcal{Y})^{2}}. $$

Thus, both solutions shown in (44) do exist if and only if the two inequalities

47 $$ \beta_{2} \le\beta\le\beta_{1} \quad\text{and} \quad\mathcal{Y} < \mathcal{Y}_{2} $$

do hold. The EE corresponding to $\widetilde{y} = {y}_{2}$ is $E_{2} = ({x}_{2}, {y}_{2}, {z}_{2})^{\prime}$ and that corresponding to $\widetilde {y} = {y}_{3}$ is $E_{3} = ({x}_{3}, {y}_{3}, {z}_{3})^{\prime}$, where $x_{i} = \widetilde{x}\vert_{\widetilde{y} = y_{i}}$ and $z_{i} = \widetilde{z}\vert _{\widetilde{y} = y_{i}}$ for $i = 2, 3$. Therefore, we state the following proposition.

#### Proposition 12

*The endemic equilibria*$E_{2}$*and*$E_{3}$*coexist if and only if the condition* (47) *holds*.

**Figure 4:**
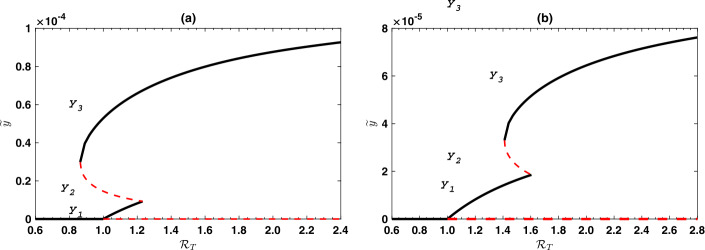
Bifurcation diagram showing the endemic prevalence of infection as a function of the effective reproduction number with treatment $\mathcal{R}_{T}$, if condition (43) holds (i.e., $\mathcal{Y} < \mathcal{Y}_{2}$). Solid curves correspond to stable equilibria and broken curves correspond to unstable equilibria. Part (**a**) shows the case where $\beta_{2} < \beta_{0}$ with $\mathcal{Y} = 0.3\mathcal{Y}_{2}$, while part (**b**) shows the case $\beta_{0} < \beta_{2}$ with $\mathcal{Y} = 0.6 \mathcal{Y}_{2}$. Simulations have been done with the parameter values shown in Table 1.

**Figure 5:**
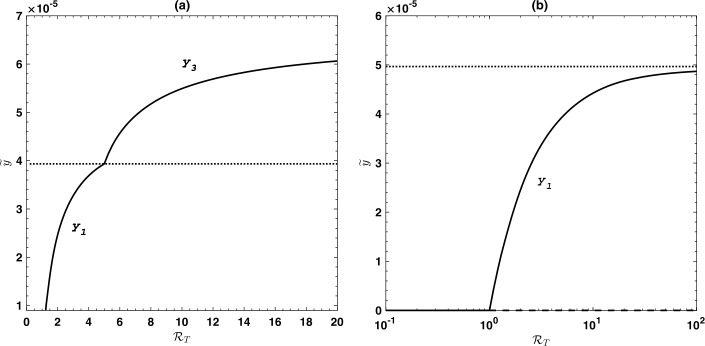
Bifurcation diagram showing the endemic prevalence of infection as a function of the effective reproduction number with treatment $\mathcal{R}_{T}$ for values of $\mathcal{Y} \ge \mathcal{Y}_{2}$. Part (**a**) shows the case where $\mathcal{Y}_{2} <\mathcal{Y} < \mathcal{Y}_{1}$ ($\mathcal{Y} = 0.8 \mathcal{Y}_{1}$), while part (**b**) shows the case $\mathcal{Y} > \mathcal{Y}_{1}$ ($\mathcal{Y} = 1.01 \mathcal{Y}_{1}$). The horizontal dotted straight line correspond to $\widetilde{y} = \mathcal{Y}$. Simulations have been done with the parameter values shown in Table 1.

Figure 4 shows the endemic prevalence of infection *ỹ* as a function of the effective reproduction number in the presence of treatment $\mathcal{R}_{T}$, for the case where $\mathcal{Y} < \mathcal {Y}_{2}$. The figure shows the three solutions $y_{1}$, $y_{2}$ and $y_{3}$. The case $\mathcal{Y}_{2} \le\mathcal{Y} < \mathcal{Y}_{1}$ means a forward bifurcation at the point $\mathcal{P}_{1}$ exists. The bifurcation program corresponding to this case is shown in Fig. 5(a). If $\mathcal{Y} \ge\mathcal{Y}_{1}$, then the point $\mathcal {P}_{1}$ does not exist and Eq. (39) has no solution. Therefore, the corresponding bifurcation diagram is drawn and shown in Fig. 5(b). It is worth noting that at the points $\mathcal{P}_{1}$ and $\mathcal{P}_{2}$ a saddle-node bifurcation starts to appear, where two equilibria collide and mutually annihilate.

The endemic prevalence of infection corresponding to the equilibrium $E_{3}$ is given by

$$\begin{aligned} {y}_{3} = \frac{- B + \sqrt{B^{2} - 4 A C}}{2 A} = \frac{-B}{2A} + \sqrt { \biggl( \frac{B}{2A} \biggr)^{2} -\frac{C}{A}}. \end{aligned}$$

It is worth noting that the two coefficients *A* and *B* are functions of the contact rate *β*. Moreover, if we assume that all model parameters except *β* are fixed, then

48 $$\begin{aligned} \frac{\partial y_{3}}{\partial\beta} = & \frac{1}{2 \sqrt{ ( \frac {B}{2A} )^{2} -\frac{C}{A}}} \biggl( \biggl({ \frac{B}{A} - 2 \sqrt{ \biggl( \frac{B}{2A} \biggr)^{2} -\frac{C}{A}} } \biggr) \frac{\partial}{\partial \beta} \biggl(\frac{B}{2A} \biggr) + \frac{C}{A^{2}} \frac{\partial A}{\partial \beta} \biggr) \\ = & \frac{1}{2A^{2} \sqrt{ ( \frac{B}{2A} )^{2} -\frac{C}{A}}} \biggl( \biggl({ \frac{-B}{2A} + \sqrt{ \biggl( \frac{B}{2A} \biggr)^{2} -\frac{C}{A}} } \biggr) \biggl( B \frac{\partial A}{\partial\beta} - A \frac{\partial B}{\partial\beta} \biggr) + C \frac{\partial A}{\partial\beta} \biggr) \\ = & \frac{1}{2A^{2} \sqrt{ ( \frac{B}{2A} )^{2} -\frac{C}{A}}} \biggl( \biggl( B \frac{\partial A}{\partial\beta} - A \frac{\partial B}{\partial\beta} \biggr) {y}_{3} + C \frac{\partial A}{\partial\beta} \biggr). \end{aligned}$$

On the other hand, we have

$$\begin{aligned} B = & \bigl( \mu (\gamma+ \mu) - P_{r} \mu \alpha \mathcal{Y} - (1-P_{r}) (\mu- \alpha\mathcal{Y}) q_{0} \beta \bigr) \\ = & \mu \bigl( \gamma+ \mu- (1 - P_{r}) q_{0} \beta \bigr) + \alpha \mathcal{Y} \bigl( (1 - P_{r}) q_{0} \beta- P_{r} \mu \bigr) \\ = & \mu \bigl( \gamma+ \mu- (1 - P_{r}) q_{0} \beta \bigr) + \frac {\alpha \mathcal{Y} }{\gamma+ \mu} A \end{aligned}$$

and

$$ \frac{\partial A}{\partial\beta} = (1 - P_{r}) q_{0} (\gamma+ \mu), \qquad \frac{\partial B}{\partial\beta} = \frac{\alpha \mathcal{Y} }{\gamma + \mu} \frac{\partial A}{\partial\beta} - (1 - P_{r}) q_{0} \mu. $$

Hence,

$$ B \frac{\partial A}{\partial\beta} - A \frac{\partial B}{\partial\beta } = (1- P_{r}) q_{0} \mu(\gamma+ \mu) (\gamma+ \mu- P_{r} \mu). $$

Therefore,

$$ \frac{\partial y_{3}}{\partial\beta} = \frac{(1- P_{r}) q_{0} \mu (\gamma+ \mu) ( (\gamma+ \mu- P_{r} \mu) {y}_{3} + \alpha \mathcal{Y} )}{2A^{2} \sqrt{ ( \frac{B}{2A} )^{2} -\frac{C}{A}}} > 0. $$

Thus, we show the following proposition.

#### Proposition 13

*The endemic prevalence of infection*${y}_{3}$*corresponding to the equilibrium*$E_{3}$*is monotonically increasing in the contact rateβ*.

It is easy to check that

$$ \lim_{\beta\to\infty}{\frac{B}{2A}} = \frac{-(\mu- \alpha\mathcal {Y})}{2(\gamma+ \mu)} \quad\text{and} \quad \lim_{\beta\to\infty }{ \frac{C}{A}} = 0. $$

Therefore,

$$ \lim_{\beta\to\infty}{y_{3}} = \frac{-B}{2A} + \biggl\vert \frac{B}{2A} \biggr\vert = \frac{(\mu- \alpha\mathcal{Y})}{2(\gamma+ \mu)} + \frac {(\mu- \alpha\mathcal{Y})}{2(\gamma+ \mu)} = \frac{\mu- \alpha \mathcal{Y}}{\gamma+ \mu}. $$

Hence, we show the following proposition.

#### Proposition 14

*The upper bound of the endemic prevalence of infection in the presence of treatment is given by*

49 $$ y_{3}^{\max} = \frac{\mu- \alpha\mathcal{Y}}{\gamma+ \mu}. $$

### The critical contact rate $\beta^{\star}$

The critical contact rate [22–24], denoted by $\beta^{\star}$, is a threshold value of the contact rate at which positive endemic states start to appear; i.e., it separates between non-existence and existence of positive persistent solutions. If $\beta< \beta^{\star}$, then the infection dies out, while if $\beta\ge\beta^{\star}$, then the infection persists. Mathematically,

50 $$ \beta^{\star} = \left \{ \textstyle\begin{array}{l@{\quad}l} \min(\beta_{0}, \beta_{2}) & \text{if } 0 < \mathcal{Y} < \mathcal {Y}_{2}, \\ \beta_{0} & \text{if otherwise}. \end{array}\displaystyle \right . $$

The first branch of (50) represents the case where multiple endemic equilibria do exist. In this case, there are two sub-cases: either $\beta_{2} < \beta_{0}$ or $\beta_{0} < \beta_{2}$.

Assume now that $\beta_{2} < \beta_{0}$. Then

$$ \sqrt{\mu(\gamma+ \mu)} + \sqrt{\alpha\mathcal{Y} \bigl(\gamma+ \mu- P_{r}(\mu- \alpha\mathcal{Y})\bigr)} < \sqrt{\frac{(\gamma+ \mu+ \alpha )(\mu- \alpha\mathcal{Y})^{2}}{\mu}}. $$

Hence,

$$ \alpha\mathcal{Y} \bigl(P_{r} \alpha\mathcal{Y} + \gamma+ (1- P_{r}) \mu \bigr) < \biggl((\mu- \alpha\mathcal{Y}) \sqrt{ \frac{\gamma+ \mu+ \alpha}{\mu}} - \sqrt{\mu(\gamma+ \mu)} \biggr)^{2}. $$

That is,

51 $$ A_{2} (\alpha\mathcal{Y})^{2} - B_{2} ( \alpha\mathcal{Y}) + C_{3} > 0, $$

where

$$\begin{aligned}& A_{2} = \gamma+ \alpha+ (1-P_{r}) \mu, \\& B_{2} = \mu \bigl( \gamma+ \alpha + (1-P_{r})\mu+ { ( \sqrt {\gamma+ \mu+ \alpha} - \sqrt{\gamma+ \mu} )}^{2} \bigr), \\& C_{2} = \mu^{2} { ( \sqrt{\gamma+ \mu+ \alpha} - \sqrt{\gamma+ \mu } )}^{2}. \end{aligned}$$

The inequality (51) is equivalent to

52 $$ \biggl(\mathcal{Y} - \frac{\mu}{\alpha} \biggr) \biggl( \mathcal{Y} - \frac {\mu}{\alpha} \times\frac{ ( \sqrt{\gamma+ \mu+ \alpha} - \sqrt {\gamma+ \mu} )^{2}}{\gamma+ \alpha+ (1-P_{r}) \mu} \biggr) > 0. $$

Since $\mathcal{Y} < \mathcal{Y}_{2} < \mu /(\gamma+ \mu+ \alpha)$, the condition (52) holds only if

53 $$ \mathcal{Y} < \frac{\mu}{\alpha} \times\frac{ ( \sqrt{\gamma+ \mu + \alpha} - \sqrt{\gamma+ \mu} )^{2}}{\gamma+ \alpha+ (1-P_{r}) \mu} : = \mathcal{Y}_{3}. $$

**Figure 6:**
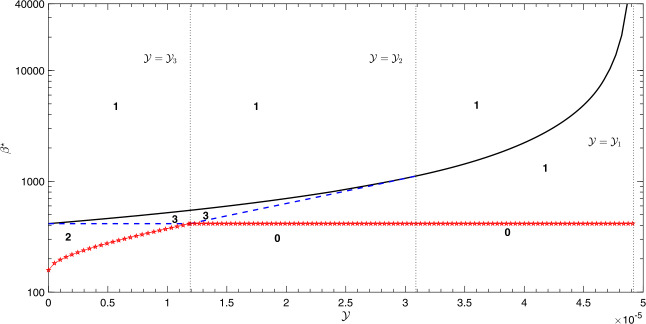
Bifurcation diagram in the plane ($\mathcal{Y}, \beta$) showing the number of endemic equilibria for the different levels of the contact rate *β* and the maximum treatment capacity $\mathcal{Y}$. The numbers “**0**, **1**, **2** and **3**” shown on the subregions denote the number of endemic equilibria in that region. The curve with pentagram represents $\beta = \beta^{\star}$ below which no endemic equilibria exist. Simulations have been done with the parameter values shown in Table 1.
- If $\mathcal{Y}_{3} \le\mathcal{Y} < \mathcal{Y}_{2}$, then $\beta_{0} < \beta_{2}$ and, therefore, $\beta^{\star} = \beta_{0}$, but the bifurcation diagram looks like that shown in Fig. 4(b) where the model shows the existence of multiple supercritical endemic steady states. In other words, no EE exists for $\beta\le\beta_{0}$, a UEE ($E_{1}$) exists for $\beta_{0} < \beta< \beta_{2}$, three endemic equilibria ($E_{1}$, $E_{2}$ and $E_{3}$) exist for $\beta_{2} \le\beta\le\beta _{1}$, while a UEE ($E_{3}$) exists for $\beta_{1} < \beta$; see Fig. 6.
- If $\mathcal{Y}_{2} \le\mathcal{Y} < \mathcal{Y}_{1}$, then $\beta _{2}$ does not exist and, therefore, $\beta^{\star} = \beta_{0}$. The bifurcation diagram looks like that shown in Fig. 5(a) where the model shows the existence of a UEE ($E_{1}$ for $\beta_{0} < \beta< \beta_{1}$ and $E_{3}$ for $\beta_{1} \le\beta$); see Fig. 6.
- If $\mathcal{Y} \ge\mathcal{Y}_{1}$, then $\beta_{1}$ is not defined and the model has a UEE ($E_{1}$) that exists for $\beta> \beta_{0}$ (see Fig. 6) and, therefore, $\beta^{\star} = \beta_{0}$. The bifurcation diagram looks like that shown in Fig. 5(b).

Hence, we have the following:

- If $0 < \mathcal{Y} <\mathcal{Y}_{3}$, then $\beta_{2} < \beta_{0}$ and, therefore, $\beta^{\star} = \beta_{2}$. The bifurcation diagram looks like that shown in Fig. 4(a) where the model exhibits the existence of multiple subcritical endemic steady states. In other words, two endemic equilibria ($E_{2}$ and $E_{3}$) exist if $\beta_{2} \le \beta< \beta_{0}$, while three positive endemic equilibria ($E_{1}$, $E_{2}$ and $E_{3}$) exist for $\beta_{0} \le\beta\le\beta_{1}$ and a UEE ($E_{3}$) exists for $\beta_{1} < \beta$; see Fig. 6.

Motivated by the above results, the following proposition is stated.

#### Proposition 15

*The critical contact rate* $\beta^{\star}$*below which the infection does not persist is given by*

54 $$ \beta^{\star} = \left \{ \textstyle\begin{array}{l@{\quad}l} \beta_{2} & \textit{if } 0 < \mathcal{Y} < \mathcal{Y}_{3}, \\ \beta_{0} & \textit{if otherwise}. \end{array}\displaystyle \right . $$

It is easy to check that

$$\begin{aligned}& \frac{\partial\beta_{0}}{\partial P_{r}} = \frac{\gamma+ \mu+ \alpha }{q_{0} (1 - P_{r})^{2}} > 0, \\& \begin{aligned}\frac{\partial\beta_{2}}{\partial P_{r}} & = \frac{\mu ( \sqrt{\mu (\gamma+ \mu)} + \sqrt{\alpha\mathcal{Y} (\gamma+ \mu- P_{r}(\mu- \alpha\mathcal{Y}))} )}{q_{0} (\mu- \alpha\mathcal{Y})^{2} (1 - P_{r})^{2}} \\ &\quad{}\times\biggl( \sqrt{\mu(\gamma+ \mu)} + \frac{\alpha\mathcal{Y} (\gamma+ \alpha\mathcal {Y})}{ \sqrt{\alpha\mathcal{Y} (\gamma+ \mu- P_{r}(\mu- \alpha\mathcal {Y}))}} \biggr) \\ & > 0.\end{aligned} \end{aligned}$$

**Figure 7:**
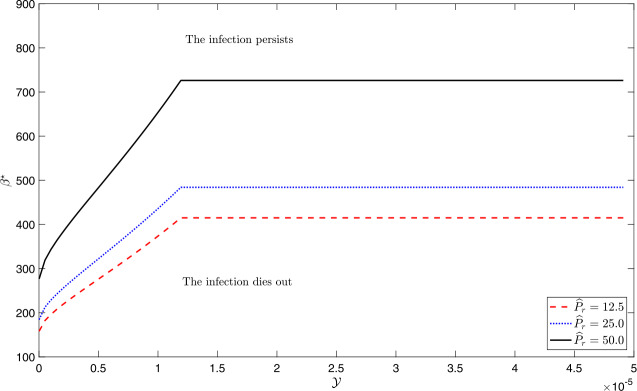
The critical successful contact rate $\beta^{\star}$ as a function of the maximum treatment capacity $\mathcal{Y}$, for different levels of percent reduction $\widehat{P}_{r}$. Below the curves, the infection does not persist, while above them the infection persists. The dashed curve is produced with a 12.5% reduction, the dotted curve is produced with 25% reduction, while the solid curve is drawn with 50% reduction in infected individuals’ contact-activity time. The values of the other parameters are as shown in Table 1. The critical contact rate $\beta^{\star}$increases with the increase of $\widehat{P}_{r}$ value.

Hence, if all parameters except $P_{r}$ have been kept fixed, then the critical contact rate $\beta^{\star}$ increases with the increase of the percent reduction proportion $\widehat{P}_{r} = 100 \times P_{r}$ which in turn extends the region of non-persistence of infection; see Fig. 7. Hence, we show the following.

#### Proposition 16

*The non*-*persistence of infection’s area extends with the increase of the percent reduction proportion*$\widehat{P}_{r}$.

### Stability analysis of the endemic equilibria $E_{2}$ and $E_{3}$ for model (31)

When ignoring the z-equation of model (31) and computing the Jacobian matrix for the remaining system at a general equilibrium $(\widetilde{x}, \widetilde{y})$ for the case $y \ge\mathcal{Y}$, where $T(y) = \alpha\mathcal{Y} = \text{constant}$, we get

55 $$ J = \left ( \textstyle\begin{array}{c@{\quad}c} -{\mu} - \frac{(1-P_{r}) q_{0} \beta \widetilde{y}}{1 - P_{r} \widetilde {y}} & - \frac{(1-P_{r})q_{0} \beta \widetilde{x}}{(1 - P_{r} \widetilde {y})^{2}}\\ \frac{(1-P_{r}) q_{0} \beta \widetilde{y}}{1 - P_{r} \widetilde{y}} & \frac {(1-P_{r})q_{0} \beta \widetilde{x}}{(1 - P_{r} \widetilde{y})^{2}} - (\gamma + \mu) \end{array}\displaystyle \right ). $$

Hence,

$$ \det{J} = (\gamma+ \mu) \biggl( \mu+ \frac{(1-P_{r}) q_{0} \beta \widetilde{y}}{1 - P_{r} \widetilde{y}} \biggr) - \mu \frac{(1-P_{r}) q_{0} \beta \widetilde{x}}{(1 - P_{r} \widetilde{y})^{2}}. $$

From the equilibrium y-equation of model (31) we have

$$ \frac{(1-P_{r}) q_{0} \beta \widetilde{x}}{1 - P_{r} \widetilde{y}} = \frac {\alpha\mathcal{Y}}{\widetilde{y}} + (\gamma+ \mu). $$

Hence,

$$\begin{aligned} \det(J) = & (\gamma+ \mu) \biggl( \mu+ \frac{(1-P_{r}) q_{0} \beta \widetilde{y}}{1 - P_{r} \widetilde{y}} - \frac{\mu}{1 - P_{r} \widetilde {y}} \biggr) - \frac{\mu\alpha\mathcal{Y}}{\widetilde{y} (1 - P_{r} \widetilde{y})} \\ = & \frac{1}{\widetilde{y}(1 - P_{r} \widetilde{y})} \bigl( (\gamma+ \mu) \bigl((1-P_{r}) q_{0} \beta- \mu P_{r} \bigr) {\widetilde{y}}^{2} - \mu \alpha\mathcal{Y} \bigr) \\ =& \frac{1}{\widetilde{y}(1 - P_{r} \widetilde{y})} \bigl(A {\widetilde {y}}^{2} - C \bigr) = \frac{1}{\widetilde{y}(1 - P_{r} \widetilde{y})} (- B {\widetilde{y}} - 2 C ) \\ =& \frac{-1}{\widetilde{y}(1 - P_{r} \widetilde{y})} \biggl( \frac{B(-B \mp\sqrt{B^{2} - 4 AC})}{2A} + 2 C \biggr) \\ =& \frac{-1}{2 A \widetilde{y}(1 - P_{r} \widetilde{y})} \bigl( -\bigl(B^{2} - 4 A C\bigr) \mp \sqrt{B^{2} - 4 AC} \bigr) \\ =& \frac{B^{2} - 4 AC}{2 A \widetilde{y}(1 - P_{r} \widetilde{y})} \biggl( 1 \pm\frac{B}{\sqrt{B^{2} - 4 AC}} \biggr). \end{aligned}$$

Since $B < 0$ and ${\vert B \vert} /{\sqrt{B^{2} - 4 A C }} > 1$,

$$ 1 + \frac{B}{\sqrt{B^{2} - 4 A C }} < 0 \quad\text{and} \quad1 - \frac {B}{\sqrt{B^{2} - 4 A C }} > 0. $$

The first expression corresponds to $y_{2} = (-B - \sqrt{B^{2} - 4 A C})/(2A)$, while the second expression corresponds to $y_{3} = (-B + \sqrt {B^{2} - 4 A C})/(2A)$. Thus,

56 $$ \det(J_{E_{2}}) < 0, \quad\text{while } \det(J_{E_{3}}) > 0. $$

It implies that the EE $E_{2}$ is unstable. Hence, we show the following proposition.

#### Proposition 17

*The EE*$E_{2}$*of model* (31) *is unstable whenever it exists*.

To complete the stability investigation of the equilibrium $E_{3}$, it remains to check the sign of the trace of *J* computed at $E_{3}$. From the equilibrium y-equation of (31) when $T(y) = \alpha \mathcal{Y}$ we have

57 $$ \frac{(1-P_{r}) q_{0} \beta\widetilde{x}\widetilde{y}}{1 - P_{r} \widetilde {y}} = \alpha \mathcal{Y} + (\gamma+ \mu) \widetilde{y}. $$

Hence,

$$ \begin{aligned}\operatorname{tr}(J) & = -(\gamma+ 2\mu) - \frac{(1-P_{r}) q_{0} \beta \widetilde{y}}{1 - P_{r} \widetilde{y}} + \frac{(1-P_{r})q_{0} \beta \widetilde{x}}{(1 - P_{r} \widetilde{y})^{2}} \\ & = - (\gamma+ 2\mu) - .\frac{(1-P_{r}) q_{0} \beta \widetilde{y}}{1 - P_{r} \widetilde{y}} + \frac{ \alpha \mathcal{Y} + (\gamma+ \mu )\widetilde{y}}{\widetilde{y} (1 - P_{r} \widetilde{y})} \\ & = \frac{\mu \alpha \mathcal{Y} - \mu^{2} \widetilde{y} + \mu (P_{r}(\gamma+ 2\mu) - (1-P_{r}) q_{0} \beta ) {\widetilde{y}}^{2} }{\mu \widetilde{y} (1 - P_{r} \widetilde{y})}.\end{aligned} $$

From (39) and (45) we have

$$ \mu\alpha\mathcal{Y} = C = - A {\widetilde{y}}^{2} - B \widetilde{y}. $$

Hence,

$$\begin{aligned} \operatorname{tr}(J) = & \frac{1}{\mu \widetilde{y} (1 - P_{r} \widetilde{y})} \bigl( - A { \widetilde{y}}^{2} - B \widetilde{y} - \mu^{2} \widetilde{y} + \mu \bigl( P_{r}(\gamma+ 2\mu) - (1-P_{r}) q_{0} \beta \bigr) {\widetilde {y}}^{2} \bigr) \\ = & \frac{-1}{\mu(1 - P_{r} \widetilde{y})} \bigl( \mu^{2} + B + \bigl( A + (1-P_{r}) q_{0} \beta\mu- P_{r} \mu( \gamma+ 2\mu) \bigr) \widetilde {y} \bigr). \end{aligned}$$

At the equilibrium point $E_{3}$ we have $\widetilde{y} = \widetilde {y_{3}}$ with $B = - 2 A \widetilde{y_{3}} + \sqrt{B^{2} - 4 A C }$. Hence,

58 $$\begin{aligned} \operatorname{tr}(J_{E_{3}}) = & \frac{-1}{\mu(1 - P_{r} \widetilde{y_{3}})} \bigl( \mu^{2} + B + \bigl( A + (1-P_{r}) q_{0} \beta\mu- P_{r} \mu(\gamma+ 2\mu ) \bigr) \widetilde{y_{3}} \bigr) \\ = & \frac{-1}{\mu(1 - P_{r} \widetilde{y_{3}})} \bigl( \mu^{2} + \sqrt{B^{2} - 4 A C } + \bigl( (1-P_{r}) q_{0} \beta\mu- P_{r} \mu(\gamma+ 2\mu) - A \bigr) \widetilde{y_{3}} \bigr) \\ = & \frac{-1}{\mu(1 - P_{r} \widetilde{y_{3}})} \bigl( \mu^{2} + \sqrt{B^{2} - 4 A C } - \bigl( (1-P_{r}) q_{0} \beta\gamma+ P_{r} \mu^{2} \bigr) \widetilde{y_{3}} \bigr) \\ = & \frac{-1}{\mu(1 - P_{r} \widetilde{y_{3}})} \bigl( \mu^{2} (1 - P_{r} \widetilde{y_{3}})+ \sqrt{B^{2} - 4 A C } - (1-P_{r}) q_{0} \beta\gamma \widetilde{y_{3}} \bigr). \end{aligned}$$

**Figure 8:**
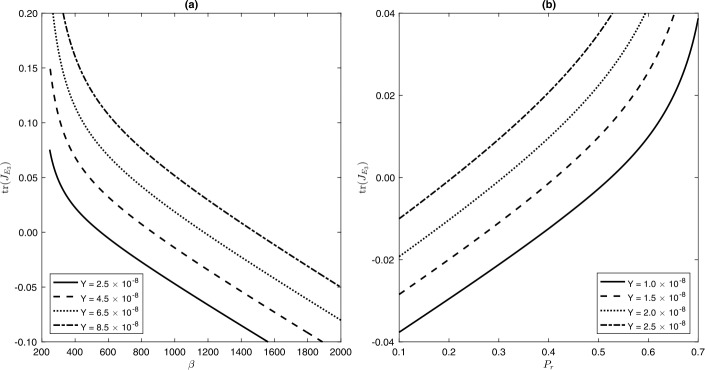
The trace of the Jacobian matrix evaluated at the EE $\operatorname{tr} J(E_{3})$ given by (63) as a function of the contact rate *β* for $P_{r} = 0.15$ (part (**a**)) and as a function $P_{r}$ for $\beta = 600$ per year (part (**b**)) and for different values of $\mathcal{Y}$, while keeping the parameters $q_{0}$, *μ*, *α* and *γ* as in Table 1.

The expression $\mu^{2} (1 - P_{r} \widetilde{y_{3}})+ \sqrt{B^{2} - 4 A C } - (1-P_{r}) q_{0} \beta\gamma \widetilde{y_{3}}$, as long as $\widetilde {y_{3}}$ is defined, could be negative. Therefore, $\operatorname{tr}(J_{E_{3}})$ could be negative and could also be positive; see Fig. 8. As long as $\operatorname{tr}(J_{E_{3}})$ is negative, the EE $E_{3}$ is locally stable. However, for a range of parameter values, where $\operatorname{tr}(J_{E_{3}})$ changes its sign to be positive, this EE loses its stability and a periodic solution may appear/disappear locally, which means that a Hopf bifurcation (i.e., a pair of complex conjugate eigenvalues crosses the imaginary axis) may exist.

### Hopf bifurcation existence

A Hopf bifurcation, also known as Poincaré–Andronov–Hopf bifurcation, is a local bifurcation in which a limit cycle is born from an equilibrium point that loses its stability, as a pair of complex conjugate eigenvalues, of the Jacobian matrix evaluated at that equilibrium, becomes purely imaginary when a model parameter crosses a critical value. For a non-linear system of two first order ordinary differential equations, whose Jacobian matrix computed at the equilibrium *E* is $J_{E}$, the characteristic equation reads

59 $$ \rho^{2} - \operatorname{tr}(J_{E}) \rho+ \det(J_{E}) = 0 $$

where *ρ* is the eigenvalue. If $\det(J_{E}) > 0$, while $\operatorname {tr}(J_{E})$ switches its sign when some model parameter (say *b*) crosses a critical level (say $b^{\star}$), then the surface of the Hopf bifurcation is given by $\operatorname{tr}(J_{E}) = 0$. Thus, the necessary and sufficient conditions for the occurrence of Hopf bifurcation are

60 $$ \operatorname{tr}(J_{E})\bigg\vert _{b = b^{\star}} = 0 \quad\text{and} \quad \frac{d \operatorname{tr}(J_{E})}{db}\bigg\vert _{b = b^{\star}} \neq0. $$

Consequently, the Hopf bifurcation surface of model (31) is given by $\operatorname{tr}(J_{E_{3}}) = 0$. Hence, with the help of (45) and (58), the Hopf bifurcation surface is given by

61 $$ \mu^{2} + B + \bigl( A + (1-P_{r}) q_{0} \beta\mu- P_{r} \mu(\gamma+ 2\mu) \bigr) \widetilde{y_{3}}= 0. $$

Now, we use (44) in (61) to get

62 $$\begin{aligned} 0 = & 2 \mu^{2} A + B \bigl(A - (1-P_{r}) q_{0} \beta\mu+ P_{r} \mu(\gamma + 2\mu) \bigr) \\ &{}+ \bigl( A + (1 - P_{r}) q_{0} \beta\mu- P_{r} \mu(\gamma+ 2\mu) \bigr) \sqrt{B^{2} - 4 A C}. \end{aligned}$$

Now, we use (45) in (62) to get

63 $$\begin{aligned} 0 = & \bigl(\mu(\gamma+ 3\mu- P_{r} \alpha\mathcal{Y}) - (1-P_{r}) (\mu - \alpha\mathcal{Y}) q_{0} \beta \bigr) \bigl((1 - P_{r}) (\gamma+ \mu) q_{0} \beta+ P_{r} \mu^{2} \bigr) \\ &{}- 2 P_{r} \mu^{3} (\gamma+ 2 \mu) + \bigl( (1-P_{r}) q_{0} \beta(\gamma+ 2 \mu)- P_{r} \mu (2\gamma+ 3\mu) \bigr) \sqrt{B^{2} - 4 AC}, \end{aligned}$$

where

$$\begin{aligned} B^{2} - 4AC &= (\mu- \alpha\mathcal{Y})^{2} (1 - P_{r})^{2} (q_{0} \beta)^{2} \\ & \quad{}- 2 \mu \bigl((\mu+ \alpha\mathcal{Y}) (\gamma+ \mu) - P_{r} \alpha \mathcal{Y} (\mu- \alpha\mathcal{Y}) \bigr) (1 - P_{r}) (q_{0} \beta) \\ &\quad{}+ \mu^{2}(\gamma+ \mu+ P_{r} \alpha \mathcal{Y})^{2}.\end{aligned} $$

If we assume that $P_{r}$ is the bifurcation parameter, then, on keeping all other model parameters fixed and letting $P_{r}$ change, a Hopf bifurcation occurs at a critical value of $P_{r}$ (say, $P_{r}^{\star}$) at which $\operatorname{tr}(J_{E_{3}})$ switches its sign; see Fig. 8(b). The figure shows that the critical level $P_{r}^{\star }$ decreases with the increase of the maximum treatment capacity $\mathcal{Y}$. It is worth noting that $P_{r}^{\star}$ is the solution of the non-linear algebraic Eq. (63). On the other hand, if *β* is considered a bifurcation parameter, while keeping $P_{r}$ and the other parameters fixed, then Fig. 8(a) shows that the critical level of the contact rate *β* at which a Hopf bifurcation exists increases with the increase of the maximum capacity treatment $\mathcal{Y}$. The following proposition summarizes the above result.

#### Proposition 18

*In the presence of a maximum capacity treatment*, *the SIR model* (31) *exhibits Hopf bifurcation whose surface is determined through* (63). *Moreover*, *on considering the reduction proportion’s parameter*$P_{r}$*as a bifurcation parameter*, *then the critical value of*$P_{r}$*at which Hopf bifurcation exists increases with the decrease of the maximum treatment capacity*$\mathcal{Y}$.

## Summary, conclusion and future work

Mathematical epidemic models have been extensively used to describe the transmission dynamics of infectious diseases, especially on the population level [1, 2, 6, 7, 9, 10, 12, 20]. As directly transmitted infections transmit from infected to susceptible (irrespective of the degree of susceptibility) individuals, it is important to model the interaction between infected and non-infected individuals in the population. The term describing this interaction is called “*incidence term*”. In some models, this term has been assumed either in mass-action [1, 5] or standard incidence form [3, 10, 12]. In other models, it is assumed in a saturated incidence [4, 19, 29] or a Holling-type form [26]. Other non-linear incidence forms are also considered in the literature [15]. Here, motivated by an SIR model for a demographically stationary population, a new incidence function that takes into account heterogeneity between infected and non-infected individuals has been introduced, differentiating between the time activity of infected and non-infected individuals in the population. Equilibrium, stability and cross-sectional analyses of the model have been done. Also, the possibility to contain an infection represented by the model with a strategy based either on vaccinating a proportion *p* of newborns or on treating infected individuals has been studied.

In the case of herd immunity, an exact formula for the critical vaccination coverage level $p_{c}$ required to eliminate the infection has been computed in terms of the percent reduction in contact time activity of infected individuals $\widehat{\mathcal{P}}$. Throughout the analysis, we concluded that the higher the percent reduction in infected individuals contact-activity time is, the lower the critical vaccination coverage required to eliminate the infection is (i.e., the higher the percent reduction in the critical vaccination coverage required to eliminate the infection is) which in turn reduces the cost of vaccination needed to protect the population from the infection.

As it is assumed that the infection is treatable, the model has been extended to include the application of a control strategy based on treating infected individuals at rate *α* with the assumption that it is possible to mostly treat a proportion $\mathcal{Y}$ of individuals. Equilibrium and stability analyses of the model have been carried out. Throughout its equilibrium analysis, the model has been shown to exhibit the existence of multiple subcritical and supercritical endemic steady states for certain range of model parameters, which means that it is possible for the infection to persist even if the effective reproduction number (in the presence of treatment $\mathcal{R}_{T}$) has been reduced to slightly below one. Motivated by the work in [23] and [24], an exact formula for the critical contact rate $\beta^{\star}$ separating between non-existence and existence of endemic infection has been computed. The analysis shows that increasing the percent reduction proportion $\widehat{P}_{r}$ increases the value of the critical contact rate $\beta^{\star}$ which in turn extends the region of non-persistence of infection and therefore reduces the infection’s minimum elimination effort $\mathcal{R} = \beta/\beta^{\star}$ [23].

In analyzing the stability of equilibria for the model with treatment, it has been shown that the EE with higher prevalence of endemic infection (denoted by $E_{3}$) loses its local stability when the trace of its Jacobian matrix switches its sign if a set of model parameters satisfy certain condition. This condition defines the Hopf bifurcation surface. On considering the parameter $P_{r}$ (which denotes the time-activity reduction proportion) as a bifurcation parameter and keeping all other model parameters fixed, the model has been shown to exhibit a Hopf bifurcation when $P_{r}$ crosses a certain (critical) level (denoted $P_{r}^{\star}$). Numerical simulations show that the higher the maximum treatment capacity ($\mathcal{Y}$) is, the lower the value of the critical $P_{r}^{\star}$ is.

Our approach could be used to formulate a more biologically meaningful model to other respiratory infectious diseases, including (but not limited to) tuberculosis, pertussis and coronaviruses. For example, if some biological factors like the inclusion of exposed and asymptomatic epidemiological states are taken into account, then a suitable model to the case of covid-19 that spreads globally in the mean time [21] could be formulated, analyzed and be deployed to have insights on the transmission dynamics and controllability of the infection. Moreover, these models could be extended to consider fractional order formulation; see for example Refs. [13, 14, 16–18].

## Appendix 1: Properties of model (5) (proof of Proposition 1)

It is easy to check that the set *Ω* is positively invariant and attracts all solutions in ${\mathbf{R}}_{+}^{3}$, as $\frac{dN}{dt} = \frac{dS}{dt} + \frac{dI}{dt} + \frac{dR}{dt} = 0$. Thus, $N(t)$ is constant. Now, to show that, for any nonnegative initial conditions $(S(0), I(0), R(0)) \in\varOmega$, the solution set $(S(t), I(t), R(t))$ of the system (5) remain positive for all $t >0$, we consider the equation

$$ \frac{dS}{dt} = \mu N - \frac{(1-P_{r}) q_{0} \beta S(t) I(t)}{N - P_{r} I(t)} - \mu S \ge - \biggl( \frac{(1-P_{r}) q_{0} \beta I(t)}{N - P_{r} I(t)} + \mu \biggr) S. $$

On separating the variables and integrating from 0 to *t* we get

$$ S(t) \ge S(0) e^{- \int_{0}^{t} { ( \frac{(1-P_{r}) q_{0} \beta I(u)}{N - P_{r} I(u)} + \mu )} \,du} \ge0. $$

Similarly, with the use of *I* and *R* equations of model (5) , we have

$$ \frac{dI}{dt} \ge-(\gamma+ \mu) I \quad\text{and} \quad \frac{dR}{dt} \ge - \mu R. $$

Hence,

$$ I(t) \ge I(0) e^{-(\gamma+ \mu)t} \ge0 \quad\text{and} \quad R(t) \ge R(0) e^{- \mu t} \ge0. $$

Thus, by comparison theorem $S(t)$, $I(t)$ and $R(t)$ are nonnegative for all $s(0), I(0) , R(0) \ge0$. Hence, given $(S(0), I(0), R(0)) \in \varOmega$, the solution set $(S(t), I(t), R(t))$ of the system (5) remain nonnegative for all $t >0$.

Now, assume that

$$\begin{aligned}& f_{1}(S, I, R) = \mu N - \frac{(1-P_{r}) q_{0} \beta S(t) I(t)}{N(t) - P_{r} I(t)} - \mu S, \\ & f_{2}(S, I, R) = \frac{(1-P_{r}) q_{0} \beta S(t) I(t)}{N(t) - P_{r} I(t)} - (\gamma+ \mu) I, \\ & f_{3}(S, I, R) = \gamma I - \mu R. \end{aligned}$$

Since *N* is constant and $N - P_{r} I(t) > 0$, it is easy to check that $f_{i}(S, I, R)$ is continuously differentiable with respect to *S*, *I*, *R* and that $\vert\frac{\partial f_{i}}{\partial x_{j}} \vert< \infty $, for all $i, j \in\{1, 2, 3\}$ and $x_{1} = S$, $x_{2} = I$ and $x_{3} = R$. Hence, the right hand side of (5) is locally Lipschitz. Therefore, there exists a unique local solution $S(t)$, $I(t)$, $R(t)$ to (5) with the initial data $S(0)$, $I(0)$, $R(0)$ on a maximum forward interval of existence [8, 11].

## Appendix 2: Local stability analysis of the endemic equilibrium $E_{1}$ for model (31)

To establish the local stability analysis for the endemic equilibrium $E_{1}$, we ignore the z-equation in model (31) and compute the Jacobian matrix for the remaining system at $E_{1}$ as

64 $$ J_{E_{1}} = \left ( \textstyle\begin{array}{c@{\quad}c} -{\mu} - \frac{(1-P_{r}) q_{0} \beta y_{1}}{1 - P_{r} y_{1}} & - \frac {(1-P_{r})q_{0}\beta x_{1}}{(1 - P_{r} y_{1})^{2}}\\ \frac{(1-P_{r}) q_{0} \beta y_{1}}{1 - P_{r} y_{1}} & \frac{(1-P_{r})q_{0}\beta x_{1}}{(1 - P_{r} y_{1})^{2}} - (\gamma+ \mu+ \alpha) \end{array}\displaystyle \right ). $$

Hence,

$$ \det(J_{E_{1}}) = (\gamma+ \mu+ \alpha) \biggl( \mu+ \frac{(1-P_{r}) q_{0} \beta y_{1}}{1 - P_{r} y_{1}} \biggr) - \mu\times\frac{(1-P_{r}) q_{0} \beta x_{1}}{(1 - P_{r} y_{1})^{2}}. $$

Using Eqs. (35), we get

$$\begin{aligned} \det(J_{E_{1}}) =& (\gamma+ \mu+ \alpha) \biggl( \mu+ \frac{(1-P_{r}) q_{0} \beta y_{1}}{1 - P_{r} y_{1}} - \frac{\mu}{1 - P_{r} y_{1}} \biggr) \\ =& \frac{\gamma+ \mu+ \alpha}{1 - P_{r} y_{1}} \times \bigl( (1-P_{r}) q_{0} \beta- \mu P_{r} \bigr) y_{1} \\ =& \frac{\gamma+ \mu+ \alpha}{1 - P_{r} y_{1}} \times\frac{\mu}{\gamma + \mu+ \alpha} \times \bigl( (1-P_{r}) q_{0} \beta- (\gamma+ \mu+ \alpha ) \bigr) \\ =& \frac{\mu(\gamma+ \mu+ \alpha)}{1 - P_{r} y_{1}} \times(\mathcal {R}_{T} - 1). \end{aligned}$$

Similarly, we use Eqs. (35) to get

$$\begin{aligned} \operatorname{tr}(J_{E_{1}}) =& -\mu- (\gamma+ \mu+ \alpha) - \frac{(1-P_{r}) q_{0} \beta y_{1}}{1 - P_{r} y_{1}} + \frac{(1-P_{r}) q_{0} \beta x_{1}}{(1 - P_{r} y_{1})^{2}} \\ = & -\mu- (\gamma+ \mu+ \alpha) - \frac{(1-P_{r}) q_{0} \beta y_{1}}{1 - P_{r} y_{1}} + \frac{\gamma+ \mu+ \alpha}{1 - P_{r} y_{1}} \\ = & -\mu- (\gamma+ \mu+ \alpha) - \frac{(1-P_{r}) q_{0} \beta y_{1} - (\gamma+ \mu+ \alpha)}{1 - P_{r} y_{1}} \\ = & -\mu - \frac{ ((1-P_{r}) q_{0} \beta - P_{r}(\gamma+ \mu+ \alpha ) ) y_{1}}{1 - P_{r} y_{1}} \\ = & -\mu - \frac{(\gamma+\mu+ \alpha)y_{1}}{1 - P_{r} y_{1}} \times \biggl( \frac{(1 - P_{r}) q_{0} \beta}{\gamma+ \mu+ \alpha} - P_{r} \biggr) \\ < & -\mu - \frac{(\gamma+\mu+ \alpha)y_{1}}{1 - P_{r} y_{1}} \times \biggl( \frac{(1 - P_{r}) q_{0} \beta}{\gamma+ \mu+ \alpha} - 1 \biggr) \\ < & -\mu - \frac{(\gamma+\mu+ \alpha)y_{1}}{1 - P_{r} y_{1}} \times ( \mathcal{R}_{T} - 1 ). \end{aligned}$$

From (36) $E_{1}$ exists if and only if

$$ 1 < \mathcal{R}_{T} < \frac{1 - P_{r} \mathcal{Y} D_{I}^{T} / L_{0}}{1 - \mathcal{Y} }. $$

Hence, $\det(J_{E_{1}}) > 0$ and $\operatorname{tr}(J_{E_{1}}) < 0$. Therefore, the endemic equilibrium $E_{1}$ for model (31) is locally asymptotically stable whenever it exists.

## Appendix 3: Derivation of Eq. (46) for $\beta_{2}$

$\beta_{2}$ is the minimum value of *β* for which the discriminant $D = B^{2} - 4 A C$ is nonnegative, where *A*, *B*, *C* are defined in (45). Therefore, $0 \le B^{2} - 4 A C$. That is,

65 $$\begin{aligned} 0 \le& (\mu- \alpha\mathcal{Y})^{2} (1 - P_{r})^{2} (q_{0} \beta)^{2} \\ &{}- 2 \mu \bigl((\mu+ \alpha\mathcal{Y}) (\gamma+ \mu) - P_{r} \alpha \mathcal{Y} (\mu- \alpha\mathcal{Y}) \bigr) (1 - P_{r}) (q_{0} \beta)+ \mu ^{2}(\gamma+ \mu+ P_{r} \alpha\mathcal{Y})^{2}. \end{aligned}$$

With some computations, one may show that

66 $$\begin{aligned} & \mu^{2} \bigl((\mu+ \alpha\mathcal{Y}) (\gamma+ \mu) - P_{r} \alpha \mathcal{Y} (\mu- \alpha\mathcal{Y}) \bigr)^{2} - \mu^{2} (\mu- \alpha \mathcal{Y})^{2} (\gamma+ \mu+ P_{r} \alpha\mathcal{Y})^{2} \\ &\quad= 4\mu^{3} \alpha\mathcal{Y}(\gamma+ \mu) \bigl( \gamma+ \mu+ P_{r}(\alpha\mathcal{Y} - \mu) \bigr) \\ &\quad= 4 \mu^{2} \bigl( \mu(\gamma+ \mu ) \bigr) \bigl( \alpha\mathcal{Y} \bigl( \gamma+ \mu- P_{r} (\mu- \alpha \mathcal{Y}) \bigr) \bigr). \end{aligned}$$

On the other hand,

67 $$\begin{aligned} (\mu+ \alpha\mathcal{Y}) (\gamma+ \mu) - P_{r} \alpha\mathcal{Y} ( \mu - \alpha\mathcal{Y}) = \mu(\gamma+ \mu) + \alpha\mathcal{Y} \bigl( \gamma+ \mu- P_{r}(\mu- \alpha\mathcal{Y}) \bigr) . \end{aligned}$$

Using (66) and (67), the inequality (65) could be rewritten as

$$ \bigl(\beta- \beta^{-}\bigr) \bigl(\beta- \beta^{+} \bigr) > 0, $$

where $\beta^{-} < \beta^{+}$ and

$$\begin{aligned}& \beta^{-} = \frac{\mu{ ( \sqrt{\mu(\gamma+\mu)} - \sqrt{\alpha \mathcal{Y} ( \gamma+ \mu- P_{r}(\mu- \alpha\mathcal{Y}) )} )}^{2}}{q_{0} (1-P_{r}) (\mu- \alpha\mathcal{Y})^{2}}, \\& \beta^{+} = \frac{\mu{ ( \sqrt{\mu(\gamma+\mu)} + \sqrt{\alpha \mathcal{Y} ( \gamma+ \mu- P_{r}(\mu- \alpha\mathcal{Y}) )} )}^{2}}{q_{0} (1-P_{r}) (\mu- \alpha\mathcal{Y})^{2}} = \beta_{2}. \end{aligned}$$

Therefore, $B^{2} - 4 A C \ge0$ if and only if $\beta\ge\beta_{2}$ and the proof is complete.
